# Therapeutic Responses to Combination Nivolumab and Temozolomide as Salvage Therapy for Metastatic Melanoma: A Case Series

**DOI:** 10.1093/oncolo/oyad184

**Published:** 2023-06-20

**Authors:** Rachel S Goodman, Seungyeon Jung, Jessica Quintos, Douglas B Johnson

**Affiliations:** Department of Hematology/Oncology, Vanderbilt University School of Medicine, Nashville, TN, USA; Department of Hematology/Oncology, Vanderbilt University School of Medicine, Nashville, TN, USA; Department of Hematology/Oncology, Vanderbilt University School of Medicine, Nashville, TN, USA; Department of Hematology/Oncology, Vanderbilt University Medical Center, Nashville, TN, USA

**Keywords:** melanoma, metastatic melanoma, immunotherapy, chemotherapy, temozolomide, nivolumab, salvage therapy

## Abstract

The management of metastatic melanoma patients that fail multiple lines of systemic therapy remains a significant challenge. There is limited literature regarding combination of anti-PD-1 and temozolomide, or of other chemotherapy agents, in melanoma. Here, we present a series of 3 patients with metastatic melanoma and their responses to nivolumab and temozolomide combination therapy after progression on several local/regional therapies, combination immune checkpoint inhibitors, and/or targeted therapies. The novel combinatory strategy led to remarkable responses in all 3 patients shortly after initiating treatment with tumor remission and symptomatic improvement. The first patient has had ongoing response 15 months after initiating treatment, although he has since discontinued temozolomide due to intolerance. The remaining 2 patients show ongoing response after 4 months, with good tolerability. This case series suggests that nivolumab and temozolomide may be a promising option in the setting of advanced melanoma refractory to standard treatments, and warrants further investigation in larger series.

## Teaser

Given the limited therapeutic options for patients who have disease progression after multiple lines of systemic therapy, there is growing promise in novel combinatorial strategies. This case series suggests that nivolumab and temozolomide may be a promising option in the setting of advanced melanoma refractory to standard treatments and warrants further investigation in larger series.

## Introduction

Immune checkpoint inhibitors (ICIs) have transformed the treatment of advanced melanoma, but many patients fail to respond or develop secondary resistance. Treating these patients, especially those lacking BRAF V600 mutations, remains challenging as standard treatments are suboptimal. Combining chemotherapy and immunotherapy (CIT) has shown promise in non-small cell lung cancer,^1,2^ with limited evidence in melanoma.^3^ We describe 3 cases of metastatic melanoma patients who responded to nivolumab/temozolomide combination after progressing on anti-PD-1 combinations and targeted therapies.

## Case Description

### Case 1

A 19-year-old male presented with BRAF V600K-mutated stage IIID melanoma of the scalp (T4b [ulcerated, Breslow thickness 4.5 mm], N3a [7/48 lymph nodes], M0). After resection, he began adjuvant ipilimumab/nivolumab given the near-certainty of residual micro-metastatic disease, which was complicated by myositis after cycle 1. Adjuvant dabrafenib/trametinib were discontinued after 12 days due to grade 3 (CTCAE v5.0) rash and fevers. After nodal progression, he started encorafenib/binimetinib but progressed after 9 months (bone, further skin/lymph node). He then received talimogene laherperpvec (T-VEC) for 3 doses and subsequently was treated with ipilimumab/nivolumab and concurrent radiation, all with rapid progression.

**Figure 1.**
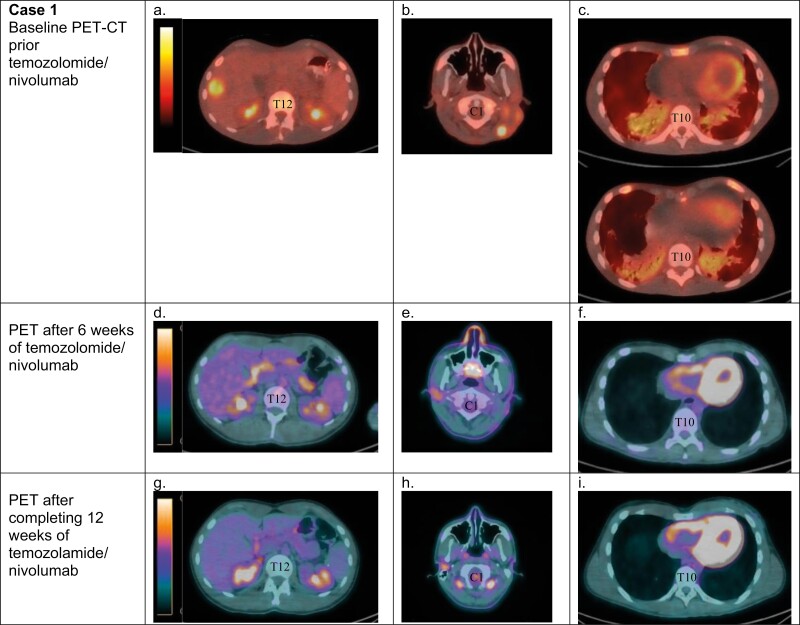
Case 1, 19-year-old male with metastatic melanoma. Baseline PET-CT prior to temozolomide/nivolumab showed new FDG avid segment 5/6 hepatic metastasis (**a**), increased FDG avid left neck subcutaneous and cutaneous metastases (**b**), and new FDG avid bilateral lower lobe consolidations (**c**). After 6 weeks of temozolomide/nivolumab, the liver metastasis decreased in size and no longer demonstrated uptake above background (**d**), the cutaneous/subcutaneous lesions in the left neck decreased in size and intensity of uptake with only mild residual uptake (**e**), and there was interval resolution of bilateral lower lobe consolidations (**f**). After 12 weeks of temozolomide/nivolumab, there were no suspicious FDG avid lesions in the abdomen or pelvis (**g**), no FDG avid cervical lymph nodes (h), and no FDG avid or otherwise suspicious pulmonary nodules (**i**).

He had 40 lb weight loss, ECOG performance status of 3, LDH level of 1352 units/L, and widespread liver, bone, cutaneous, and nodal metastases. He began nivolumab (240 mg q2 weeks) and temozolomide (200 mg/m2, 5 of 28 days), with normalization of LDH and rapid improvement in functional status. Re-staging PET-CTs after 6 weeks of treatment demonstrated a significantly decreased number and intensity of cutaneous/subcutaneous and skeletal lesions with a resolution of liver metastases (Fig. 1). The liver lesion decreased by 70% in size. He experienced a grade 3 hypersensitivity reaction with neuropathy, intermittent fevers, itching, and headache 20 min after taking temozolomide, leading to discontinuation after the 3rd cycle. He continued nivolumab monotherapy for an additional 6 months and has had an ongoing partial response on PET-CTs (with complete metabolic response) approximately 15 months after treatment.

### Case 2

**Figure 2.**
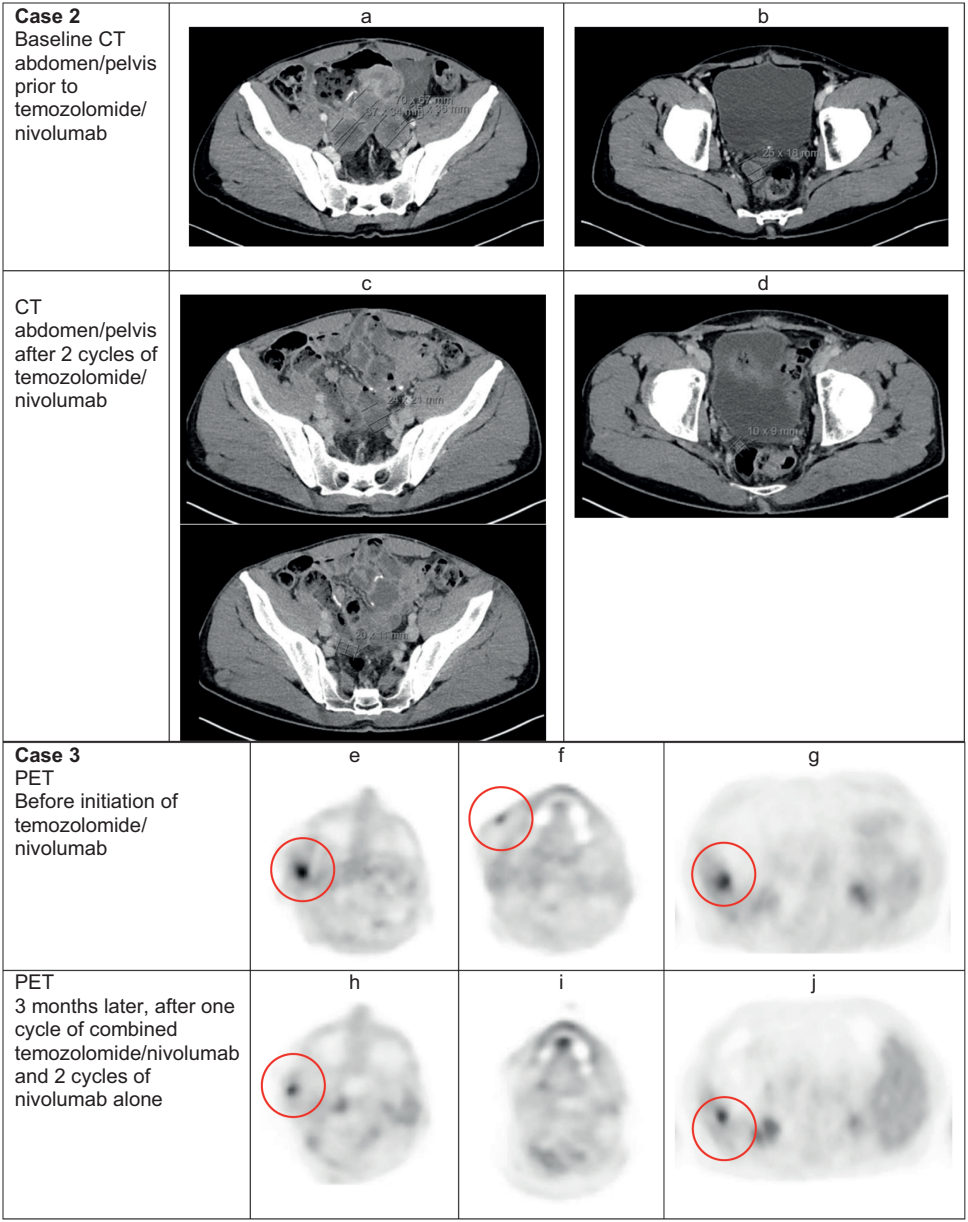
Case 2, 38-year-old male with metastatic melanoma. (**a**) Prior to treatment, there were enlarging multilobulated centrally hypodense masses within the pelvis. In its entirety, the mass measured 7 × 6.7 cm. (**c**) After treatment, the multilobulated mass decreased in size with one mass measuring 2.4 × 2.1 cm (top image) and the second mass measuring 2 × 1.1 cm (bottom image). (**b**) Prior to treatment, a new 2.5 × 1.8 cm central low attenuation nodule was noted in the pelvis. (**d**) After treatment, this nodule in the pelvis decreased in size to 1 × 0.9 cm. Case 3, 78-year-old male with metastatic melanoma. **(e, h)** There is decreased size with similar metabolic activity of involvement in the right pre-auricular and parotid region after initiation of nivolumab and temozolomide. The size decreased from **(e)** 2.2 × 1.4 cm with maximal SUV of 8.5 to **(h)** 1 cm with maximal SUV of 7.9. **(f, g)** The focus of activity along the anterior margin of the right facial reconstructive changes resolved after treatment with nivolumab and temozolomide. **(g, j)** There was a decrease in size (from 1.6 cm to 1.2 cm) but similar activity (from SUV 6.7 to 6.0) of the metastatic right hepatic lobe lesion after nivolumab/temozolomide.

A 38-year-old male presented with intermittent abdominal pain and was diagnosed with BRAF V600D mutant primary mucosal melanoma of the small bowel. Five months post-resection, CT demonstrated small bowel thickening consistent with metastatic disease, and he was started on encorafenib/binimetinib. Seven months after initially demonstrating response, he developed new small bowel metastases and received 4 cycles of ipilimumab/nivolumab before further small bowel and mesenteric progression. Four months after resection and reinitiation of encorafenib/binimetinib, he experienced further progression and underwent another segmental small bowel resection. Afterward, he received an experimental dendritic cell therapy, high-dose vitamin infusion, and nivolumab (480 mg q4 weeks), but progressed 3 months later with additional peritoneal and intestinal lesions resected. After further progression, he began temozolomide (200 mg/m2, 5 of 28 days), while continuing nivolumab. After 5 cycles of temozolomide with nivolumab, CT showed a partial response with a 71% decrease in the pelvic mass (Fig. 2). The patient has been tolerating the treatment well after 6 months and additional 15% tumor shrinkage (−86% by RECIST).

### Case 3

A 78-year-old male with a history of large granulocytic leukemia (LGL) with chronic pancytopenia presented with wild-type BRAF melanoma of the right cheek. 1-2 months post-resection, he developed multiple in-transit metastases and initiated pembrolizumab with progression. He began ipilimumab/nivolumab for 4 cycles with further progression with cutaneous, nodal, and biopsy-confirmed liver metastases. He then underwent radiation therapy (dose/Fx: 400 cGy, total dose 4800 cGy), ablation of hepatic lesions, followed by T-VEC to right face lesions.

After further progression, he started nivolumab (480 mg q4 weeks)/temozolomide (200 mg/m2, 5 of 28 days). Temozolomide was held after the first cycle due to worsening pre-existing chronic neutropenia (baseline ANC 1.17 × 10^3^/×L). PET-CT after 4 months showed a minor response with shrinkage in the skin, soft tissue, and hepatic lesions, and continued temozolomide at a reduced dose (200 mg/m2, 3 of 28 days) (Fig. 2). Unfortunately, PET-CT 3 months later showed progression and the patient has opted to start pembrolizumab/lenvatinib.

## Discussion and Conclusion

To our knowledge, this is the first case series of 3 consecutive patients with notable clinical responses to combination of nivolumab/temozolomide.

Emerging evidence suggests chemotherapy augments the immunologic effect of ICIs by disrupting immunosuppressive pathways and sensitizing tumor cells to T-cell-mediated killing.^4,5^ It is unclear if these effects are specific to temozolomide specifically (as compared with other chemotherapy agents), and further research is needed to determine the relative efficacy of different chemotherapeutic regimens in combination with ICIs. Clinical trials of CIT, particularly in NSCLC, have shown survival benefits and acceptable safety profiles.^1,2,6,7^ Although temozolomide is used relatively frequently for refractory advanced melanoma due to its more favorable side effect profile, it is associated with low response rates (~10%), similar to dacarbazine.^8,9^ Overall, these 3 patients, who were ineligible for clinical trials (due to deteriorating performance status, anemia/refusal, and neutropenia/pancytopenia, respectively), had good tolerability, though one patient required discontinuation due to intolerance (unclear if allergic versus hypersensitivity reaction) and another required dose reduction due to chronic neutropenia.

There is limited study of CIT in melanoma. A retrospective study of CIT in 60 melanoma patients with progression after anti-PD-1 suggested improved outcomes with CIT (median overall survival [OS] 3.5 years, 95% CI 1.7-NR) than with ICI or chemotherapy alone (median OS 1.8 years, 95% CI 0.9-2) [*P* = .02].^3^ However, only 1 patient (3%) received a combination PD-1/temozolomide with unclear response. In this series, patients with BRAF wild-type melanoma had greater benefits than those with BRAF mutant melanoma. As most BRAF mutant patients have already received targeted therapy, resistance to targeted therapy was proposed to cause cross-resistance to subsequent CIT. Two patients received both PD-1 and BRAF/MEK inhibitors prior to CIT, but prior targeted therapy did not appear to cause resistance to nivolumab/temozolomide. A single-center study also suggested the benefit of combination pembrolizumab/temozolomide as front-line treatment for advanced melanoma demonstrating higher objective response rates (ORR) (41.7%) than temozolomide/DTIC (5%) or anti-PD-1 alone (20.7%).^10^

While combination anti-PD-1/temozolomide as a salvage therapy has shown signs of promise for melanoma, there is limited literature regarding its application in real-life clinical settings. These cases offer suggestions of wider benefits for this combination and warrant further investigation.

## Data Availability

The data underlying this article will be shared on reasonable request to the corresponding author.
